# Quantitative Susceptibility Mapping Using the Multiple Dipole-inversion Combination with k-space Segmentation Method

**DOI:** 10.2463/mrms.mp.2016-0062

**Published:** 2017-03-27

**Authors:** Ryota Sato, Toru Shirai, Yo Taniguchi, Takenori Murase, Yoshitaka Bito, Hisaaki Ochi

**Affiliations:** 1 Research and Development Group, Hitachi Ltd., 1-280 Higashi-koigakubo, Kokubunji, Tokyo 185-8601, Japan; 2 Healthcare Business Unit, Hitachi, Ltd., Tokyo, Japan

**Keywords:** quantitative susceptibility mapping, vein, k-space, regularization

## Abstract

Quantitative susceptibility mapping (QSM) is a new magnetic resonance imaging (MRI) technique for noninvasively estimating the magnetic susceptibility of biological tissue. Several methods for QSM have been proposed. One of these methods can estimate susceptibility with high accuracy in tissues whose contrast is consistent between magnitude images and susceptibility maps, such as deep gray-matter nuclei. However, the susceptibility of small veins is underestimated and not well depicted by using the above approach, because the contrast of small veins is inconsistent between a magnitude image and a susceptibility map. In order to improve the estimation accuracy and visibility of small veins without streaking artifacts, a method with multiple dipole-inversion combination with k-space segmentation (MUDICK) has been proposed. In the proposed method, k-space was divided into three domains (low-frequency, magic-angle, and high-frequency). The k-space data in low-frequency and magic-angle domains were obtained by L1-norm regularization using structural information of a pre-estimated susceptibility map. The k-space data in high-frequency domain were obtained from the pre-estimated susceptibility map in order to preserve small-vein contrasts. Using numerical simulation and human brain study at 3 Tesla, streaking artifacts and small-vein susceptibility were compared between MUDICK and conventional methods (MEDI and TKD). The numerical simulation and human brain study showed that MUDICK and MEDI had no severe streaking artifacts and MUDICK showed higher contrast and accuracy of susceptibility in small-veins compared to MEDI. These results suggest that MUDICK can improve the accuracy and visibility of susceptibility in small-veins without severe streaking artifacts.

## Introduction

Quantitative susceptibility mapping (QSM) is a new magnetic resonance imaging (MRI) technique for noninvasively estimating the magnetic susceptibility of biological tissue. The estimated susceptibility of biological tissue quantifies clinically relevant values, such as tissue iron concentration^1^ or venous oxygen saturation level,^2,3^ which are expected to be applied to diagnosis or study of neurodegenerative^4,5^ or cerebrovascular diseases.^6^

As for QSM, a susceptibility map is reconstructed from a phase image acquired by a gradient echo sequence. The relationship between phase and susceptibility is simply expressed in k-space because a phase is a point-wise multiplication of susceptibility and a dipole kernel. However, direct inversion of a dipole kernel causes severely amplified noise in a reconstructed susceptibility map because the values of the dipole kernel are almost zero around the magic angle in k-space.^7^ Although this problem can be solved by acquiring an additional phase image in different magnetic-field directions,^8,9^ this solution needs more than one direction, and the head position of the subject must be rotated in each direction.

To reconstruct a susceptibility map in one acquisition, many methods have been proposed,^8,10–19^ including threshold-based k-space division (TKD)^8,10^ and morphology enabled dipole inversion (MEDI)^13,14^

TKD truncates the value of the dipole kernel around the magic angle in k-space and directly inverts the truncated dipole kernel. It can calculate a susceptibility map quickly, but streaking artifacts occur in the susceptibility map because of the truncation in k-space.

MEDI uses a priori structural information and imposes sparsity by using L1-norm regularization on the edges of a reconstructed susceptibility map that are not consistent with those in the structural information. A magnitude image is used as structural information because it can be obtained simultaneously with the phase image. Since the contrasts of some tissues, such as the deep-gray-matter nucleus, in the magnitude image agree well with those in a susceptibility distribution, MEDI can calculate an artifact-free susceptibility map of these tissues with high accuracy. However, it often fails to depict small veins because some small-vein contrasts in a magnitude image are low and the susceptibilities of these veins are underestimated due to the smoothing effects of L1-norm regularization.

Generally, L1-norm regularized susceptibility map often underestimates susceptibility of tissues with subtle susceptibility such as small veins although a susceptibility map can be calculated without streaking artifacts. On the other hand, the unregularized susceptibility map can estimate subtle susceptibility more accurately compared to L1-norm regularization although some streaking artifacts occur in the map.

In this study, to obtain a susceptibility map with improved estimation and visibility of small-vein susceptibility without severe streaking artifacts, a new method for QSM is proposed called multiple dipole-inversion combination with k-space segmentation (MUDICK). In MUDICK, k-space is divided into three domains (low-frequency, magic-angle, and high-frequency). The k-space data in low-frequency and magic-angle domains were obtained by L1-norm regularization using structural information of a pre-estimated susceptibility map. The k-space data in high-frequency domain were obtained from the pre-estimated susceptibility map in order to preserve small-vein contrasts. The pre-estimated susceptibility map is calculated without regularization because contrasts of the small-vein are well preserved. MUDICK as the proposed method was compared with the conventional methods by a numerical simulation and a human-brain study in terms of artifacts and both accuracy and visibility of small-vein susceptibility.

## Materials and Methods

### Processing flow of the proposed method

**Fig 1.**
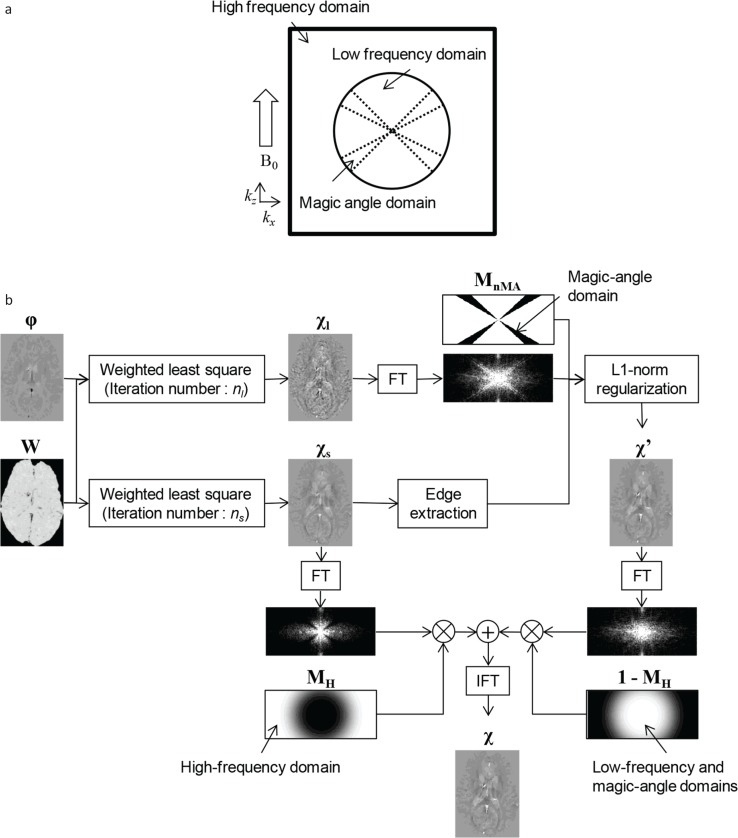
Illustrations of the proposed method. (**a**) Schematic view of k-space (in the plane of *k*_y_ = 0) with the proposed method. (**b**) Processing flow of susceptibility calculation by the proposed method. FT, fourier transformation; IFT, inverse fourier transformation.

A schematic view of k-space used by the proposed method is shown in Fig. 1a. k-space is divided into three domains (low-frequency, magic-angle, and high-frequency). In the low-frequency and magic-angle domains, L1-norm regularization is performed using an unregularized susceptibility map as structural information to suppress streaking artifacts and preserve susceptibility of small veins. The L1-norm regularization is performed with a focus on the magic-angle domain in order to apply regularization focused on an ill-conditioned domain as previously reported.^15^ In the high-frequency domain, the unregularized susceptibility map is used to further reduce underestimation of sub-voxel veins which are barely extracted as structural information. The boundaries between low- and high-frequency domains are smoothly connected using a sigmoid function in order to reduce ringing artifacts.

The processing flow of the proposed method is shown in Fig. 1b. First, unregularized susceptibility maps **χ_l_** and **χ_s_** are pre-calculated from the following equations with a weighted least-square method, respectively,

(1) $$\chi_{l}={\text{arg}\;\text{min}}_{\chi_{l}}{\left\|W\left(F^{-1}DF\chi_{l}-\delta \right)\right\|}_{2}^{2},$$

(2) $$\chi_{s}={\text{argmin}}_{\chi_{s}}{\left\|W\left(F^{-1}DF\chi_{s}-\delta \right)\right\|}_{2}^{2},$$

where **W** denotes a diagonal weighting matrix; **F** and **F**^−1^ denote three-dimensional (3D) Fourier and inverse Fourier transform operators; respectively, **D** denotes a diagonal matrix where diagonal element *d*(***k***) = 1/3 − *k_z_*^2^/(*k_x_*^2^ *+ k_y_*^2^ *+ k_z_*^2^) expresses the dipole kernel in k-space;^7^ and **δ** denotes a local field map calculated from phase image **φ**. Equations 1 and 2 are solved iteratively by using a linear-conjugate-gradient method with iteration numbers, *n_l_* and *n_s_* (*n_l_* > *n_s_*), respectively. The initial condition of **χ**_l_ and **χ**_s_ is set to a zero vector. *n_l_* is set to 50, which is large enough to converge to a stable solution. *n_s_* is chosen to maximize the visibility of small veins. Too small *n_s_* causes small veins with weak contrasts. If *n_s_* is increased too much then small veins will be depicted discontinuously due to severe noise and streaking artifacts. The unregularized susceptibility map **χ_s_** is used as structural information and high-frequency values in k-space.

Second, susceptibility map **χ’** for the low-frequency and the magic-angle domains is calculated using L1-norm regularization with structural information of **χ_s_**. L1-norm regularization is applied with a focus on the magic-angle domain in accordance with the following equation,

(3) $$\begin{array}{l} \chi^{\prime }={\text{arg}\;\text{min}}_{\chi^{\prime }}\{\frac{1}{2}{\left\|M_{nMA}F\left(\chi^{\prime }-\chi_{l}\right)\right\|}_{2}^{2}+\lambda ({\left\|M_{x}G_{x}\chi^{\prime }\right\|}_{1}\\ \;\;\;\;\;\;\;\;\;\;+{\left\|M_{y}G_{y}\chi^{\prime }\right\|}_{1}+{\left\|M_{z}G_{z}\chi^{\prime }\right\|}_{1})\}, \end{array}$$

where **M***_nMA_* denotes a diagonal binary matrix defined in k-space; *λ* denotes a regularization parameter; **M**_x_, **M**_y_, and **M**_z_ denote diagonal matrices; and **G**_x_, **G**_y_, and **G**_z_ denote gradient operators in the *x*, *y*, and *z* directions, respectively. A diagonal element in **M**_nMA_ is a binary mask defined as “1” if | *d*(***k***) | > *m_th_*, or “0” otherwise, where *m_th_* is a threshold value which defines the boundary between the low-frequency and the magic-angle domains. *m_th_* is set to 0.1 in reference to several previous studies.^2,11,19^ A diagonal element in **M**_x_ is a binary edge mask defined as “1” if |**G**_x_**χ_s_**(***r***)| < *α_s_*, or “0” otherwise, where *α_s_* is a threshold value. Diagonal elements in **M**_y_ and **M**_z_ are defined in the same manner as **M**_x_.

Lastly, susceptibility map **χ** is calculated by replacing values in the high-frequency domain of **χ’** by those of **χ_s_** in accordance with the following equation,

(4) $$\chi =F^{-1}\left\{\left(I-M_{H}\right)F\chi^{\prime }+M_{H}F\chi_{s}\right\},$$

where **M**_H_ denotes a diagonal weighting matrix for the high-frequency domain in k-space. A diagonal element in **M**_H_ (*m*_H_(***k***)) is calculated from the following sigmoid function,

(5) $$m_{\text{H}}(k)=\left[1+\text{tanh}\left\{k_{\mathrm{cur}}\left(\left|k\right|-k_{\mathrm{th}}\right)\right\}\right]/2,$$

where *k_cur_* and *k_th_* are parameters which define the smoothness and the position of boundary between the low- and the high-frequency domains, respectively. When *k_cur_* decreases, the boundary is smoothly connected but the calculated susceptibility in the deep-gray-matter nuclei will be changed.

### Numerical simulation

In the numerical simulation, artifacts, small-vein visibility, and estimation accuracy of the calculated susceptibility map were evaluated by using a susceptibility distribution model including small veins and deep-gray-matter nuclei. The obtained results by MUDICK as the proposed method were compared with those by TKD and MEDI as the conventional methods.

**Fig 2.**
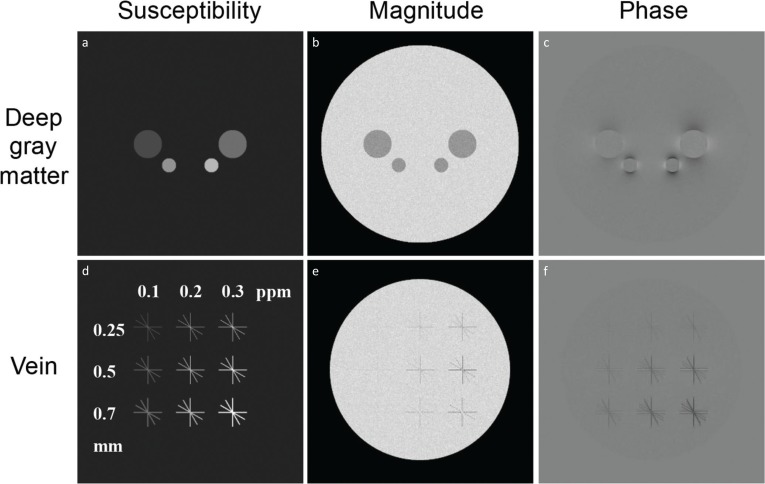
Simulation models. (**a**, **d**) Model susceptibility distribution, including four deep-gray-matter nuclei with different susceptibilities and 36 small veins with different susceptibilities, diameters and angles. (**b**, **e**) Model magnitude image. (**c**, **f**) Model phase image. Figures in the top row show slices, including deep-gray-matter nuclei, and figures in the bottom row show slices including small veins.

The susceptibility distribution model consists of 800 × 640 × 640 voxels with 0.25-mm iso-resolution. Two representative coronal slices (400th and 541st slices) of the susceptibility distribution model are shown in Fig. 2a and d. In this model, the brain was modeled as an ellipsoid (the long axis is the anterior-posterior direction) with four ellipsoids or spheres simulating the deep-gray-matter nucleus and 36 cylinders simulating small veins. The susceptibility of the brain was set to 0 ppm, and the susceptibilities of the deep-gray-matter nucleus were set to 0.05, 0.1, 0.15, and 0.2 ppm. To evaluate reconstructed susceptibility of various types of veins, the susceptibility, diameter, and angle of veins in this model were changed. The susceptibilities of veins, *χ_v_*, were set to 0.1, 0.2, and 0.3 ppm. The diameters of veins, *d_v_*, were set to 0.25, 0.5, and 0.7 mm. Note that the diameters of veins have modeling errors of up to a voxel in accordance with their position and angle due to the voxel grid. The angles between the vein axis and the B_0_ direction, *θ_v_*, were set to 0, 30, 60, and 90 degrees. In a model magnitude distribution, the pixel values were set to 1 in the brain and veins and 0.7 in the deep-gray-matter nucleus.

A phase distribution was calculated by applying a forward method^7,20^ to the susceptibility model with imaging parameters B_0_ = 3T (axial direction) and TE = 20 ms. A model magnitude image and a model phase image (400 × 320 × 320 voxels with 0.5-mm iso-resolution) were then calculated by shrinking the complex image consisting of the model magnitude distribution and the model phase distribution. Gaussian noise was added to the images under the assumption that the signal-to-noise ratio (SNR) in the brain is 30. Two representative coronal slices (200th and 271st slices) of the model magnitude and phase images are shown in Figs. 2b and e, 2c and f, respectively. For the determination of parameters and the evaluation of calculation accuracy, a reference susceptibility map was calculated by shrinking the susceptibility distribution model to 400 × 320 × 320.

In the calculation of susceptibility by the proposed method, diagonal elements of **W** in Eqs. 1 and 2 were set to values given by a binary brain mask. In the calculation of the diagonal elements in **M**_x_, **M**_y_, and **M**_z_, the threshold *α_s_* was defined as four times the standard deviation of the pre-estimated susceptibility map (**χ_s_**) in the brain region. Isolated zero points in the binary edge masks were changed to one. To improve the visibility of small veins and maintain the calculation accuracy of susceptibility, the reconstruction parameters of *λ*, *k_cur_*, *n_s_*, and *k_th_* were determined as described below. The value of *λ* in Eq. 3 was set to 10^−1.75^, which gave the minimum root-mean-square error (RMSE) between the calculated (**χ’** in Eq. 3) and the reference maps (on the condition that *λ* was incremented by a multiplication factor of 10^0.25^). *k_cur_* was set to five. This value connected the boundary between the low- and the high-frequency domains in k-space as smoothly as possible under the condition that the change of susceptibility by *k_cur_* was less than 1% in the deep-gray-matter nuclei.

To determine the optimal values of *n_s_* and *k_th_*, the dependences of small-vein visibility on *n_s_* and *k_th_* were evaluated, respectively. The small-vein visibility was evaluated by using the ratio of standard deviation to the mean value (coefficient of variation [CV]) in the region of interest (ROI) of small veins. When the calculated susceptibility distribution of a vein is close to the susceptibility distribution model, the CV of the calculated susceptibility distribution in the vein will be close to zero. The ROI was set in the small veins with minimum diameter and susceptibility (*d_v_* = 0.25, *χ_v_* = 0.1) because these veins were the most difficult to visualize in the model. The dependence of the susceptibility in the small veins with a maximum diameter (*d_v_* = 0.7) on *k_th_* was also evaluated because *k_th_* affected the accuracy of susceptibility in small veins. On the bases of the evaluated dependences, the optimal values of *n_s_* and *k_th_* were determined, respectively.

For comparison, susceptibility maps by TKD^10^ and MEDI^13,14^ methods were calculated. These methods were programmed in-house. As for the calculation of TKD, the threshold value was set to 0.18, and the values of the dipole kernel in the region where their absolute values were lower than the threshold were truncated. The threshold value was determined as the minimum point of RMSE between the calculated and reference maps under conditions in which the slope of the regression line between the maps in the deep-gray-matter nucleus was greater than 0.8. As for the calculation of MEDI, a regularization parameter (multiplied to the regularization term) was set to 10^−3.25^, which gave the minimum point of RMSE between the calculated and reference maps. Evaluation functions including the L1-norm term (Eq. 3 and MEDI) were minimized using the non-linear conjugate-gradient method.^21^

Calculations for all methods were performed using MATLAB R2015a on a Windows 7 Professional workstation with an Intel^®^ Xeon^®^ Processor E5-2623 v3 and 32 GB RAM.

To compare the conventional and proposed methods, artifacts, small-vein visibility, and accuracy for small veins were evaluated. Artifacts were evaluated visually and quantitatively in the coronal slice including deep-gray-matter nucleus. Two ROIs were set in the regions of the artifacts for quantitative evaluation. Mean of susceptibility was calculated in the ROI. Small-vein visibility was evaluated visually in the coronal slice including the veins. The calculation accuracy was evaluated by comparing the mean susceptibilities in the reference model with the calculated susceptibility map in the ROIs in veins. Nine ROIs were set for veins with different diameters *d_v_* and model susceptibilities *χ_v_*. Each ROI was defined in a region of four small veins (except the center and both ends) with the same *d_v_* and susceptibility *χ_v_* by thresholding the reference susceptibility map. One ROI for the brain region was also set in slices without any tissues.

### Human study

In the study on the human brain, streaking artifacts and small-vein susceptibility obtained by the proposed method (MUDICK) were compared with those obtained by the conventional methods (TKD and MEDI).

Scanning experiments on healthy volunteers were performed on a 3T MRI (Hitachi, Ltd. Tokyo, Japan) equipped with a 32-channel phased-array coil positioned around the head. The scan parameters in axial slices were as follows: sequence: 3D radio-frequency (RF)-spoiled gradient echo with flow compensation, repetition time (TR)/echo time (TE) = 49.4/30.0 ms; flip angle (FA) = 15; number of signal averages (NSA) = 1; band width = 13.1 kHz; acceleration factor = 3; resolution: 0.5 × 0.5 × 2.0 mm (reconstructed to 0.43 × 0.43 × 1.0 mm); and field of view (FOV): 220 × 220 × 120 mm. The scan time was 4 min and 58 sec. Data from the volunteer were obtained in accordance with the regulations of the internal review board of the Central Research Laboratory, Hitachi, Ltd., following receipt of written informed consent. The obtained phase images were unwrapped using a region-growing method, and RESHARP (regularization enabled SHARP)^22^ was performed to remove the background phase.

In the calculation of the proposed method, the diagonal element *w*(**r**) of **W** was set to the value given by a weight image calculated from the phase image as described below. First, complex image c(***r***) was calculated from the phase image as c(***r***) = exp(*j*φ(***r***)) (where *j* is an imaginary number). Next, phase-variation image v(***r***), in which each pixel value *v_i_* was a standard deviation of c(***r***) in a 3 × 3 × 3 region around pixel *i*, was calculated. Lastly, weight *w*(***r***) was calculated by using a phase-variation image as *w*(***r***) = 1−v(***r***). *n_s_*, *λ*, *k_th_*, and *k_cur_* were set to the same values as those in the numerical simulation. The threshold *α_s_* was defined as the mean value of the absolute gradient of the pre-estimated susceptibility map in the brain.

Susceptibility maps were also calculated from obtained phase images by TKD^10^ and MEDI,^13,14^ which were programmed in-house. The threshold value in the calculation of TKD and the regularization parameter in the calculation of MEDI were set to the same values as those in the numerical simulation. Calculations for all methods were performed using the same software and workstation as in the numerical simulation. To compare the proposed and the conventional methods, streaking artifacts and small-vein susceptibility were evaluated. Streaking artifacts were evaluated visually in coronal and sagittal images. Small-vein susceptibility was evaluated by image contrasts and line profiles in an axial image.

## Results

### Numerical simulation

**Fig 3.**
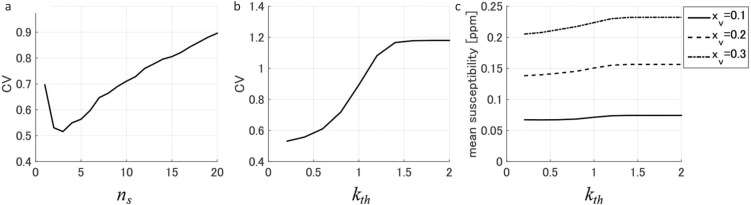
Dependences of small-vein visibility and susceptibility on reconstruction parameters (*n*_s_ and *k*_th_). (**a**) Dependence of coefficient of variation (CV) in small veins with minimum diameter and susceptibility (*d*_v_ = 0.25, x_v_ = 0.1) on *ns*. (**b**) Dependence of CV in small veins with minimum diameter and susceptibility on *k*_th_. (**c**) Dependence of mean susceptibility in small veins with maximum diameter (*d*_v_ = 0.7) on *k*_th_.

Figure 3 shows the dependences of small-vein visibility and susceptibility on the reconstruction parameters (*n_s_* and *k_th_*). As shown in Fig. 3a, the CV of the small veins with minimum diameter and susceptibility (*d_v_* = 0.25, *χ_v_* = 0.1) was minimized when *n_s_* = 3. This result shows that the visibility of small veins was maximized when *n_s_* = 3. Therefore, *n_s_* was set to three. As shown in Fig. 3b, the CV of the small veins with minimum diameter and susceptibility (*d_v_* = 0.25, *χ_v_* = 0.1) increased with *k_th_* when *k_th_* was less than 1.4. On the other hand, as shown in Fig. 3c, mean susceptibility in small veins with maximum diameter (*d_v_* = 0.7) slightly decreased with decreasing *k_th_*. These results show that there is a trade-off between the visibility in small veins with minimum diameter and the accuracy of calculated susceptibility in small veins with maximum diameter. On the basis of these results, *k_th_* was set to 0.6 in order to maximize the visibility of small veins with minimum diameter under the condition that the rate of the decrease in susceptibility of small veins with maximum diameter (*d_v_* = 0.7) was within 10%.

**Fig 4.**
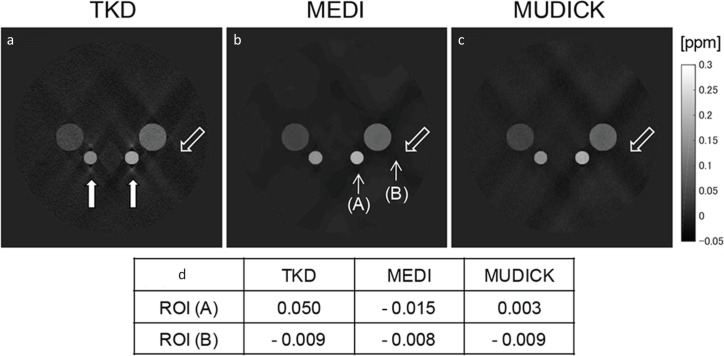
Comparison of artifacts around deep gray matter nucleus between the conventional and proposed methods in numerical simulation. (**a–c**) Susceptibility maps calculated by (**a**) thresholdbased k-space division (TKD), (**b**) morphology enabled dipole inversion (MEDI), and **(c)** multiple dipole-inversion combination with k-space segmentation (MUDICK) in slices of deep gray matter nucleus. Solid arrows in (**a**) indicate artifacts visible only in TKD, and outlined arrows in (**a–c**) indicate artifacts visible in all methods. (**d**) Quantitative comparison of artifacts. Mean susceptibilities in ppm are calculated in the two regions of interest (ROI) set as 5 × 5 pixels in the positions indicated by thin arrows in (**b**). The positions of ROI (A) and ROI (B) are selected visually to indicate the position of the above two types of artifacts.

Susceptibility maps around deep-gray-matter are shown in Fig. 4a–c. As shown by solid arrows in Fig. 4a, severe artifacts were visible around the deep-gray-matter nucleus with high susceptibility in the TKD susceptibility map. As shown by outlined arrows in Fig. 4a–c, weak artifacts were visible around the large deep-gray-matter nucleus in all susceptibility maps. These artifacts were compared quantitatively in Fig. 4d. In ROI (A), absolute mean susceptibility calculated by MUDICK was the smallest value in all methods, and mean susceptibility calculated by TKD was the largest value, 0.050 ppm, which was comparable to susceptibility values of some deep-gray-matter nuclei. In ROI (B), mean susceptibility calculated by MUDICK was almost equal to those calculated by the conventional methods, and its absolute value was less than 0.01 ppm.

**Fig 5.**
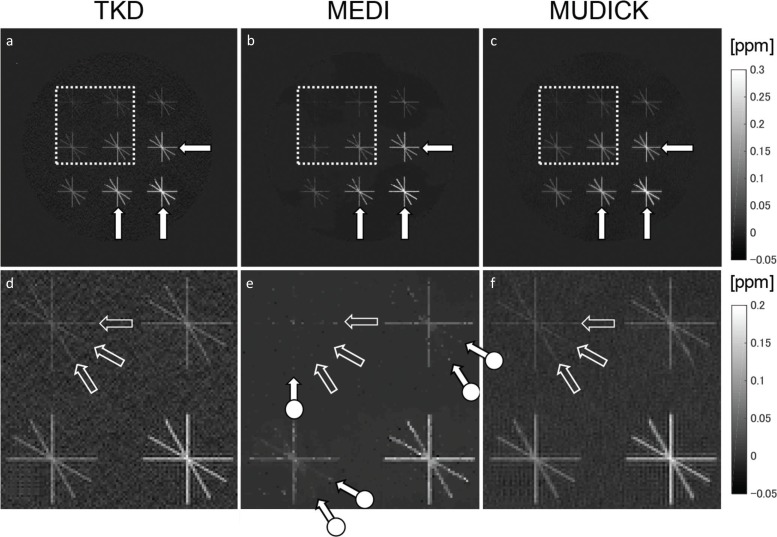
Comparison of small vein contrasts between the conventional and proposed methods in numerical simulation. (**a–c**) Susceptibility maps calculated by (**a**) thresholdbased k-space division (TKD), (**b**) morphology enabled dipole inversion (MEDI), and (**c**) multiple dipole-inversion combination with k-space segmentation (MUDICK) in slices of small veins. Solid arrows indicate veins visible in all methods. (**d–f**) Enlarged images of susceptibility maps in the regions marked as white dashed-line squares in (**a–c**). Outlined arrows indicate the positions of veins barely visible in any methods, and circle-ended arrows in (**e**) indicate positions of veins invisible in MEDI but visible in MUDICK.

Figure 5 shows small-vein contrasts calculated by TKD, MEDI, and MUDICK. As shown by the arrows in Fig. 5a–c, thick veins with high susceptibility were well depicted in susceptibility maps calculated by all methods. On the other hand, as shown in outlined arrows in Fig. 5d–f, small veins with minimum diameter and susceptibility were barely visible in any susceptibility maps. As shown in circle-ended arrows in Fig. 5e, some small veins were invisible in the MEDI susceptibility map but visible in the MUDICK susceptibility map.

**Fig 6.**
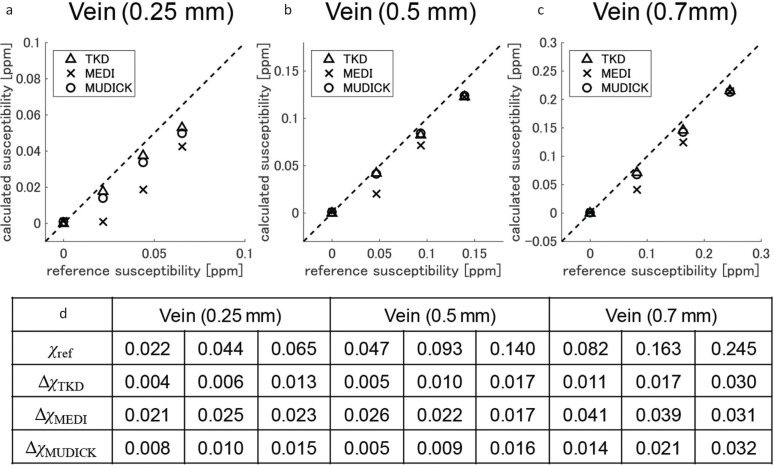
Comparison of estimation accuracy in small veins between the conventional and proposed methods. (**a–c**) Plots of mean susceptibilities in regions of interest (ROIs) of reference and calculated susceptibility maps. ROIs are set in small veins with diameters of (**a**) 0.25 mm, (**b**) 0.5 mm, and (**c**) 0.7 mm. Mean susceptibilities in brain ROI are also plotted in (**a–c**). Dashed lines in (**a–c**) indicate the lines of y = x. Note that the values of reference susceptibilities are untrue values that include partial volume effects. (**d**) Comparison of calculation errors in small veins. χ_ref_ denotes mean susceptibilities in ROIs of reference map. ΔχTKD, ΔχMEDI, and ΔχMUDICK denote calculation errors of TKD, MEDI, and MUDICK, respectively. Calculation errors are defined as the absolute difference between mean susceptibilities of reference and calculated susceptibility maps. MEDI, morphology enabled dipole inversion; MUDICK, multiple dipole-inversion combination with k-space segmentation; TKD, thresholdbased k-space division.

Comparison results of mean susceptibilities in small veins are shown in Fig. 6. As shown in Fig. 6, MUDICK showed higher small-vein susceptibility than MEDI (Fig. 6a–c) and reduced calculation errors (Fig. 6d). These results indicate higher accuracy in small-vein susceptibility. As shown in Fig. 6a–c, MEDI strongly underestimates susceptibilities in small veins with minimum diameter (Fig. 6a) or small veins with low susceptibility (cross marks away from the dashed lines in Fig. 6b and c). Although TKD showed slightly lower calculation errors than MUDICK (Fig. 6d), it showed severe artifacts as previously shown in Fig. 4a.

These results suggest that MUDICK improved visibility and estimation of small-vein susceptibility without severe artifacts.

### Human study

**Fig 7.**
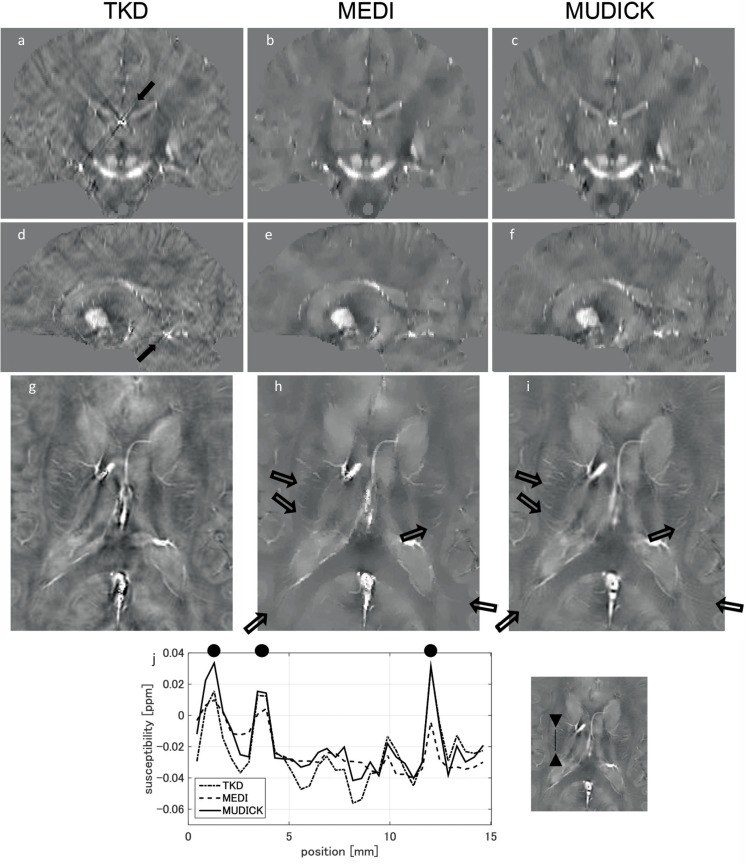
Comparison of susceptibilities calculated by conventional and proposed methods in human brain study. (**a–c**) Coronal images of susceptibility maps of (**a**) thresholdbased k-space division (TKD), (**b**) morphology enabled dipole inversion (MEDI), and (**c**) multiple dipole-inversion combination with k-space segmentation (MUDICK). (**d–f**) Sagittal images of susceptibility maps of (**d**) TKD, (**e**) MEDI, and (**f**) MUDICK. Solid arrows in (**a**) and (**d**) indicate streaking artifacts. (**g–i**) Enlarged axial images of susceptibility maps around small veins calculated by (**g**) TKD, (**h**) MEDI and (**i**) MUDICK. Outlined arrows indicate veins. (**j**) Line profile of susceptibility of veins. Position of line is indicated in right image (which is the same as (**i**). Black circles indicate positions of veins.

Coronal and sagittal images of susceptibility maps calculated by TKD, MEDI, and MUDICK are shown in Fig. 7a–c and Fig. 7d–f, respectively. The severe streaking artifacts were visible in the TKD susceptibility map, as indicated by the arrows in Fig. 7a and d, but were invisible in MEDI and MUDICK susceptibility maps. Enlarged susceptibility maps around small veins calculated by TKD, MEDI, and MUDICK are shown in Fig. 7g–i. As shown by the arrows in Fig. 7h and i, the contrasts of small veins were better in the MUDICK susceptibility map than in the MEDI susceptibility map. Line profiles of small veins are shown in Fig. 7j. In Fig. 7j, the susceptibility of small veins calculated by MUDICK (solid line) was larger than that calculated by MEDI (dashed line). These results suggest that MUDICK improved visibility and reduced underestimation of small-vein susceptibility without severe streaking artifacts.

Calculation times for each susceptibility map were 3 sec in TKD, 17 min 3 sec in MEDI, and 24 min 55 sec in MUDICK.

## Discussion

The numerical simulation and human-brain study show that the proposed method improves the estimation and visibility of small-vein susceptibility. Estimated small-vein susceptibility is improved for two reasons. One is that the unregularized susceptibility map used as structural information shows higher contrasts in some small veins than a magnitude image. When L1-norm regularization with edge masks of the unregularized susceptibility map was applied, the noise and streaking artifacts were suppressed while the susceptibility of small veins was preserved. The other reason is that high-frequency values in k-space are replaced. Even though the contrasts of the sub-voxel veins in the unregularized susceptibility map are not enough to extract edges, the proposed method can improve estimation and visibility of susceptibility in these veins by replacing high-frequency values in k-space by this map.

In the numerical simulation, as shown in circle-ended solid arrows in Fig. 5e, the veins with angles of 30 and 60 degrees (with respect to B_0_ direction) were more difficult to depict than those with angles of 0 and 90 degrees. This dependency on vein angles corresponds to the results of a simulation study by Fan et al.,^3^ which showed that the calculation error of venous susceptibility is largest when vein angle is 45 degrees with respect to B_0_ direction. As mentioned in this study, the dependency of visibility in our study is related to the absolute value of the dipole kernel in k-space. The values of the dipole kernel in k-space are nearly zero in the region around the magic angle. The veins with angles of 30 and 60 degrees have more energy in this region than those with angles of 0 and 90 degrees, which results in more underestimation of venous susceptibility. Except the small veins with minimum diameter and susceptibility, MUDICK can visualize small veins even with angles of 30 and 60 degrees more clearly than to MEDI as shown in Fig. 5e and f.

The dependences of small-vein visibility and susceptibility on *n_s_* and *k_th_* were evaluated in Fig. 3. As shown in Fig. 3a, as *n_s_* increased, the CV was firstly decreased, and then increased. It is considered that this dependency of CV on *n_s_* is caused by the dependency of contrasts and noise in small veins. In the conjugate gradient method, the contrasts rapidly increase in the first few iterations. Then, noise and streaking artifacts mainly increase in the later iterations.^23^ Therefore, if too small *n_s_* (such as *n_s_* = 1) is used, the contrasts in the small veins will be weak. On the other hand, if too large *n_s_* (such as *n_s_* = 10) is used, small veins will be discontinuously visualized by severe noise and streaking artifacts. By setting *n_s_* with minimum CV (*n_s_* = 3), the visibility of small veins is maximized with increased contrasts and suppressed noise.

In regard to *k_th_*, there was a trade-off between visibility in small veins with minimum diameter (*d_v_* = 0.25) and accuracy in small veins with maximum diameter (*d_v_* = 0.7). As *k_th_* decreased, the visibility in small veins with minimum diameter improved (Fig. 3b), but the accuracy in small veins with maximum diameter slightly decreased (Fig. 3c). The cause of this trade-off is that *k_th_* controls the size of the high-frequency domains replaced by **χ**_s_ from **χ’** using Eqs. 4 and 5. **χ_s_** is superior to **χ’** in visibility of small veins, but inferior to **χ’** in accuracy of small veins with maximum diameter. In this study, in consideration of this trade-off, *k_th_* = 0.6 was selected using criteria to allow a 10% decrease in the susceptibility of small veins with maximum diameter. If a smaller *k_th_* is used, the visibility in small veins will improve more. However, as shown in the comparison of small-vein susceptibility with other methods (Fig. 6c), susceptibility of small veins with maximum diameter and model susceptibility (*d_v_* = 0.7, *χ_v_* = 0.3) calculated by MUDICK will be smaller than those calculated by TKD or MEDI if *k_th_* is decreased. Therefore, the above criteria were used to improve the visibility of small veins with the accuracy higher than or comparable to the other methods.

Although the proposed method reduces the underestimation of small-vein susceptibility, the small-vein susceptibility in numerical simulation is still more underestimated than true susceptibility (0.1, 0.2, and 0.3 ppm), especially in the case of sub-voxel tissues, as shown in Fig. 6a. The possible reason for this underestimation is the partial-volume effect in the phase image. For tissues whose sizes are comparable to or smaller than voxel size, phase variations within and around these tissues are averaged and resulting susceptibility values are underestimated. To reduce underestimation caused by the partial-volume effect, phase image should be acquired with higher spatial resolution or susceptibility values should be corrected. This acquisition with a higher spatial resolution is a straightforward approach, but a longer scan time is needed. To correct susceptibility values, the correction coefficient, which may be determined from vein information such as diameter and angle, should be pre-calculated. Reducing underestimation caused by the partial-volume effect without using a prolonged scan time is a challenge for the future.

## Conclusion

To obtain a susceptibility map with improved estimation and visibility of small vein without severe streaking artifacts, a method was proposed that uses a pre-estimated susceptibility map without regularization as structural information and values of the high-frequency domain in k-space. A numerical simulation and a human-brain study showed that the proposed method improves estimation and visibility of small-vein susceptibility without severe streaking artifacts.
